# The Reliability of a Three-Dimensional Photo System- (3dMDface-) Based Evaluation of the Face in Cleft Lip Infants

**DOI:** 10.1155/2012/138090

**Published:** 2012-08-05

**Authors:** Rebecca Ort, Philipp Metzler, Astrid L. Kruse, Felix Matthews, Wolfgang Zemann, Klaus W. Grätz, Heinz-Theo Luebbers

**Affiliations:** ^1^Clinic for Cranio-Maxillofacial Surgery, University Hospital of Zurich, Frauenklinikstrasse 24, 8091 Zurich, Switzerland; ^2^Surgical Planning Laboratory, Harvard Medical School, Brigham and Women's Hospital, 75 Francis Street, Boston, MA 02115, USA

## Abstract

Ample data exists about the high precision of three-dimensional (3D) scanning devices and their data acquisition of the facial surface. However, a question remains regarding which facial landmarks are reliable if identified in 3D images taken under clinical circumstances. Sources of error to be addressed could be technical, user dependent, or patient respectively anatomy related. Based on clinical 3D photos taken with the 3dMDface system, the intra observer repeatability of 27 facial landmarks in six cleft lip (CL) infants and one non-CL infant was evaluated based on a total of over 1,100 measurements. Data acquisition was sometimes challenging but successful in all patients. The mean error was 0.86 mm, with a range of 0.39 mm (Exocanthion) to 2.21 mm (soft gonion). Typically, landmarks provided a small mean error but still showed quite a high variance in measurements, for example, exocanthion from 0.04 mm to 0.93 mm. Vice versa, relatively imprecise landmarks still provide accurate data regarding specific spatial planes. One must be aware of the fact that the degree of precision is dependent on landmarks and spatial planes in question. In clinical investigations, the degree of reliability for landmarks evaluated should be taken into account. Additional reliability can be achieved via multiple measuring.

## 1. Introduction

Objective evaluation of the face is challenging. Meaningful assessment by basic measurements is hindered by the complex three-dimensional (3D) anatomy of the face because of its specific but not perfect symmetry. Anthropometry, the science of measuring the characteristics of the body [1], has dealt with this problem for many decades.

Regarding the underlying bony structures, 3D evaluations based on computed tomography data have become more and more routine [2, 3]. However, no standard has developed for three-dimensional imaging of the soft tissues so far.

The state-of-the-art method for facial soft tissue evaluation and documentation is direct measurement and two-dimensional (2D) photography [4–6]. Both have immanent downsides: Direct measurements are examiner-dependent and retrospective surveys are impossible. Both of those qualities limit the use of the application in clinical follow-up studies. Two-dimensional photography can be calibrated for true-to-scale measurements, but only distances between points in the exact same plane as the photo can be measured accurately. However, there are few flat planes on the human face. Volumetric measurements or image fusion techniques are not possible when utilizing 2D photos [1, 7–12].

Modern computer technology has opened the door to the development of computed tomography [13, 14] and intraoperative computer navigation [15], two 3D-based concepts which are routinely used in modern medicine [16]. Not yet routinely utilized but also derived from the possibilities offered by modern computer technology are numerous 3D scanning devices [17] for which scientific data about their high technical accuracy exists [18–24].

 In the facial region, these scanning devices are applied in anthropometry [25–28] to correlate facial appearance and genetic disorders [29] and, of course, to assess or predict treatment outcomes [30–34]. Even more complex 3D video techniques are utilized for dynamic evaluation [35–38]. First studies have also applied software algorithms to automatically identify pathologies [39].

Obviously, it is important to evaluate the precision and reliability of new technology before applying it in clinical routines [22, 40]. This includes the proper and reliable identification of landmarks [24, 41, 42] and their validity for the given purpose [43].

Our hypothesis was that, despite the technically sufficient precision of 3D face scanning techniques with errors below 1 mm [18, 22–24], there might be regions that are much less precisely measured in general and/or which are not reliable in every measurement.

## 2. Materials and Methods 

The aim of the study was to investigate the reliability of landmark assessment utilizing virtual 3D face models of children with cleft lips (CLs) acquired under clinical circumstances with the 3dMDface imaging system. The main goal was to identify landmarks that are reliable in marking for study purposes. Later on, strategies should be developed to achieve the highest possible repeatability and precision.

Data about the technical precision of all kinds of 3D imaging systems is available in the literature [18–24]. Our specific setting is analogous to a previously published study utilizing a phantom model. The system provides a known mean global error of 0.2 mm within a range from 0.1 mm to 0.5 mm [22].

### 2.1. Patients

Six data sets of infants between six and 18 months old-all with uni- or bilateral CL-were acquired. One additional infant without any craniofacial deformity served as a control in order to identify difficulties specifically related to CL. 

All images were taken under clinical conditions, meaning for example, no special skin preparation-except drying off saliva if necessary-was performed. Also no special lightning concept away from the flash system integrated into the imaging system was utilized. Finally all pictures were taken by medical personal in the presence of at least one parent.

The study design satisfied the criteria of the local ethics committee for being exempted from individual Institutional Review Board approval. The study design thereby fulfilled the guidelines of the Declaration of Helsinki about Ethical Principles for Medical Research Involving Human Subjects.

### 2.2. Data Acquisition and Processing

**Figure 1:**
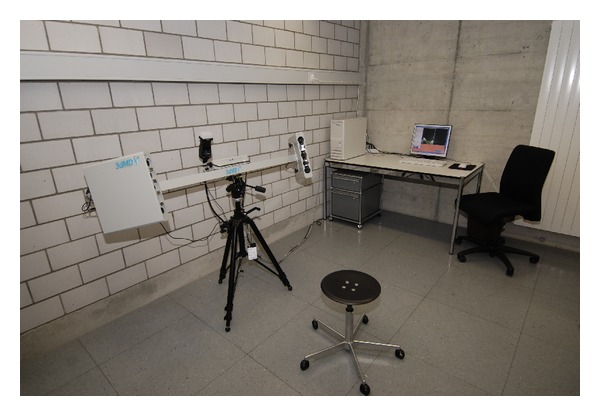
3dMDface system (3dMD Inc., Atlanta, GA, USA) as utilized for image acquisition; desktop PC performing necessary calculations and storing data.

All data were acquired with artificial lighting using the 3dMDface System (3dMD Inc., Atlanta, GA, USA) (Figure 1). Registration of the system, as recommended by the manufacturer, was performed before data acquisition. This registration process guarantees correct geometric data acquisition by software driven calibration of the camera setting. To acquire the necessary information a plate carrying a defined pattern of dots and lines is photographed in two different positions by the system, which is based on a combination of stereophotogrammetry and structured light. It acquires six pictures within 1.5 milliseconds. Four black and white images under structured light conditions were acquired for 3D surface reconstruction and two additional color images were taken for the purpose of skin surface representation. All data was calculated and stored on a desktop computer (Figure 1) attached to the system. The whole package (camera system including flashes, desktop computer, and image acquisition software) is commercially available and sold for a reasonable price compared to other medical devices. After purchasing, there are no further costs for image acquisition except the natural costs of running digital cameras and a desktop computer, mainly energy costs. Costs for a service contract-if chosen-are usually independent from the number of images acquired.

**Figure 2:**
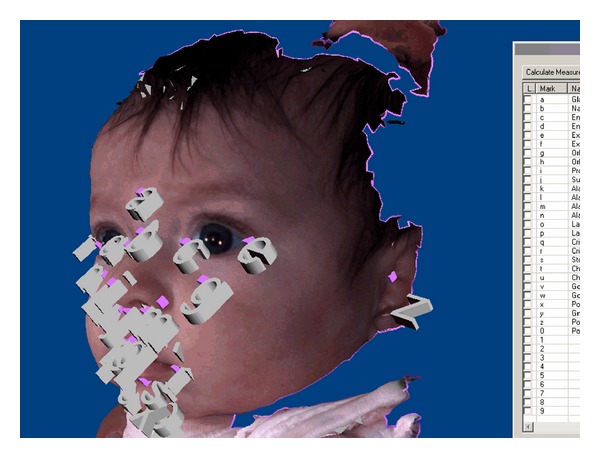
Virtual 3D model as reconstructed. (Landmarks placed and partially blocking one another as well as anatomic structures).

Each subject data acquisition was performed repeatedly until no better 3D model was practically achievable; images with obvious facial expressions were discarded. The acquisition process was considered complete if one image covered all 27 landmarks chosen for evaluation. In cases in which infants became increasingly noncompliant, the acquisition process was stopped and the image covering most of the 27 landmarks was chosen for further evaluation. An example used for a resulting virtual 3D model is shown in Figure 2.

The dataset of the chosen image was transfered to a laptop computer via USB-Stick. Further data processing was performed utilizing the 3dMD-Patient-Software (3dMD Inc., Atlanta, GA, USA) that comes with a capture device. A total of 27 landmarks were labeled on the surface of each virtual face. Landmarks were chosen due to clinical relevance and spread over the face with emphasis on aesthetically relevant regions (Figure 2). Software features such as rotation or zooming were used for the best visualization of landmarks. A list of the landmarks chosen for evaluation is presented in the first column of Table 1.

To match a typical retrospective study situation, one observer labeled all landmarks on five consecutive days and again after a break of one week. From the 3dMD-Patient-Software the *x*-, *y*-, and *z*-coordinates of these markings were directly saved into xls-files. These files then were imported into Excel for Mac 2011 (Microsoft Corporation, Redmond, WA, USA) for further analysis.

Since all measurements were performed within one virtual face model, the coordinates were identical for identical points without the necessity of superimposition or registration. Since no real truth coordinates serving as references can be derived by any means, a “mean coordinate” was calculated out of the six individual measurements. The target registration error $\left(\text{TRE}=\sqrt{\Delta x^{2}+\Delta y^{2}+\Delta z^{2}}\;\right)$ representing the three-dimensional caliper distance between this reference coordinate and each individual measurement was calculated [15, 22, 40, 44, 45].

A mean TRE for each landmark in all 3D models was calculated in order to identify landmark-specific precision. Overall, the concept of analysis was analogous to that in previously published studies concentrating on the technical precision of the system and the influence of involuntary facial movements [22, 43]. All data were analyzed using descriptive statistics. The tests were performed with SPSS 20 for Mac (SPSS Inc., Chicago, IL, USA) and Excel 2011 for Mac (Microsoft Corporation, Redmond, WA, USA).

## 3. Results and Discussion

### 3.1. Image Acquisition

A sufficient 3D image was acquired for all infants. However, it did require multiple attempts in all cases. Two to 14 (mean 7.9, standard deviation 3.9) captures were performed per child. Based on this data an image acquisition session with a child takes between 5 and 15 minutes time if measured from entering the room until leaving again. The necessary system start up and registration process (about 5 minutes) is not included and we strongly recommend to have the system ready once the child comes into the room since in most cases compliance is best at the beginning of the session and on should not waste that time spot for procedures that can be done ahead.

For the most part, incorrect head positioning was the reason for the insufficient imaging. The system's low capture time of 1.5 milliseconds guaranteed sharp images under most circumstances, but a long shutter lag sometimes allowed the children to move their faces partially out of the capture region before images were acquired. This could be resolved, for example, via a technical improvement involving a short shutter lag time. Any attempts of gently holding the head position in place mostly resulted in agitation of the infant including facial expressions leading to 3D representations that were inappropriate for craniofacial anthropometry. This issue of little children tending to move and thereby interfering with imaging is also a problem in conventional 2D photography [46] and even more so in direct anthropometry. However, technical improvements involving shorter shutter lag times could definitely help to resolve this issue.

Another common reason for necessary reacquisition was that prominent areas compromised the view of less prominent areas, resulting in poor or even missing 3D representations. Basically, this also is a problem of positioning: to achieve optimal imaging, an approximate 15-degree angled camera view from below is necessary (compare to Figure 1). The moment the infant looks downward instead of straight ahead, the nostrils block parts of the important upper lip region.

The third problem was wet skin surface areas leading to reflection and errors in 3D reconstruction. While wet skin surfaces around the nose and eyes can mostly be avoided, the presence of saliva poses a huge problem in reference to the presurgical evaluation of CL-patients. Especially in infants, it is almost impossible to dry the CL and take a 3D image before the region is covered with saliva again. Algorithms need to be developed that are robust against reflection. This might be achieved by using other light wavelengths and special algorithms to counterbalance reflection and refraction.

Until the problems of shutter lag and wet surfaces are overcome, sensitive communication with infants and accompanying adults to ensure a maximum level of compliance is the only solution. Under those conditions, a number of datasets should be captured to choose the best one at completion of the sessions. Since data volume is not an issue in reference to this technology, it is feasible to keep all of the raw data since some images might provide exact information about distinct regions despite their being of poor quality.

All of these factors point to the fact that existing highly precise data, for example, from cadaver studies [24], cannot be taken for granted when it comes to the demanding task of evaluation involving infants.

### 3.2. Data Processing

Data processing utilizing rotation and zoom of the virtual 3D model was mostly unproblematic. One drawback of the 3dMD-Patient-Software is the blocking-out of potential landmarks by another label close by and its caption, as shown in Figure 2. This problem was avoided by optimizing the sequence of marking. However, it is a clear downside for less experienced users of the software. The learning curve is unnecessarily flat due to this blocking effect. A small adjustment to the software, which allows the opportunity to hide landmarks that have already been set, would resolve the issue. The problem clearly depicts the need for user-oriented software development in reference to medical appliances. However, routine was build up quickly and labeling of the 27 landmarks took not more than 10 minutes per 3D model.

### 3.3. 3D Data

Five craniofacial landmarks (stomion, soft gonion left and right, soft porion left and right) could not be labeled on all subjects since the relevant regions were not sufficiently represented in the 3D model (compare the second column in Table 1; *N* = number of valid measurements per landmark, 42 = completed in all subjects).

Insufficiently represented landmarks appeared in five (71%) of the seven models. Only two (29%) were labeled completely. However, all other landmarks (22; 81%) other than the above mentioned (five; 19%) were accurately represented in all of the 3D models and, therefore, labeled on all subjects.

The target registration errors for each landmark are given in descriptive statistics in Table 1. The data is sorted top-down by the mean TRE for each craniofacial landmark (range 0.39 mm to 2.21 mm). The table includes the minimum, maximum, mean, standard deviation, and range for each evaluated landmark.

Even though that is not statistically significant, it is interesting to note that the control patient without CL provides the best overall accuracy. We believe this is due to the CL anatomy, which is less likely to be completely captured by the camera system. In addition the cleft region tends to be covered with saliva.

On the other hand, the differences in TRE between the best and the worst landmarks (exocanthion right, 0.39 mm; soft gonion right, 1.64 mm) are much more distinct.

Of the 27 landmarks, 21 (78%) show reliability better than onemm in mean and another four (15%) are within the range of 1–1.5 mm. Only two (7%) landmarks were revealed to be worse than 1.5 mm (Table 1). These results are conclusive in reference to the existing literature [23, 43].

**Figure 3:**
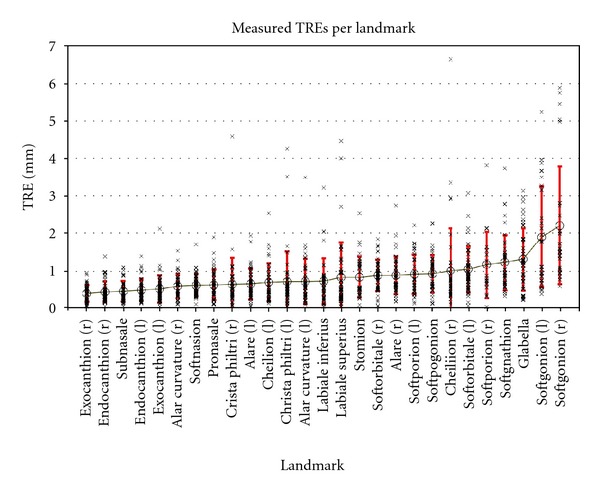
TREs per landmark (crosses); mean TRE per landmark (circle); standard deviation (red). (Sorted from best mean TRE on the left to worst on the right side).

Figure 3 outlines the range of the measurements for all landmarks in comparison to the mean and standard deviation. Even though most of the landmarks are well defined, in terms of mean values of repeated measurements, it must be stated that single measurements can easily be out of the clinically acceptable range. In general, we expect 1.5 mm to be clinically acceptable since discrepancies below 1.5 mm can not routinely be seen by the naked eye of observers [43]. However, this “rule of thumb” does not apply to landmarks that are clearly outlined by anatomical structures, for example, exo- or endocanthion. On the other hand, these clearly defined landmarks, in general, show better results when it comes to the reliability of craniofacial anthropometry, with reliability levels far below onemm (Table 1, Figure 3). To reduce the influence of outliers in reference to further studies, we recommend taking repeated measurements of the same landmark. This is analogous to the concept of building mean models to reduce the influence of involuntary mimic movements [43]. Of course, the perfect solution to the inherent lack of perfect reliability in landmark positioning among raters would be to use a fully automatic software algorithm based on objective parameters that define each landmark. However, the ability to do this seems to be technically quite far in the future.

For the soft gonion, as the least reliable landmark, it can be shown that the TRE is mainly a result of bad definition in *y*- and *z*-axes. The error in the *x*-axis, which represents the width of the mandible, is only 0.84 mm (the mean for the soft gonion right and left) compared to 1.20 mm (*y*-axis) and 1.67 mm (*z*-axis). Therefore, this landmark can very well be used to, for example, evaluate the width of the mandible. This leads to the claim that prior to any clinical study involving 3D imaging for the sake of anthropometric evaluation, the landmarks to be studied must not only be evaluated by general means but, depending on the clinical demands, precision and reliability in terms of the three special dimensions need to be analyzed in detail.

An obvious downside of this study is the small number of subjects, combined with the focus on intraobserver reliability. However, we believe this to have been overcome by the conclusive results obtained which are in line with previous studies involving model heads [22], children [23], and adults [23, 43]. Focusing on one observer within a time frame of roughly two weeks simulates a clinical study setting. More observers and more time between measurements might possibly reduce reliability but not reveal additional information about the precision of the presented technique in an assumed study setting.

The fact that only one 3D imaging system was evaluated is not a downside to the study results since the systems on the market provide more or less the same technical precision [18–24] and the same quality of surface representation. The issue would be different if a new high-resolution system providing a significantly higher image quality presumably leading to more reliable landmark identification were to come on the market.

The technique of 3D photography seems to be valid for facial soft tissue analysis. Data can be acquired under clinical conditions and, without loss of precision, retrospectively analyzed under study conditions. This is a new option for craniofacial measurements that cannot be achieved by direct anthropometric measurements or 2D photography. However, one must be aware and take into account the fact that every landmark has its own level of precision and reliability that also depends on the spatial plane in question. In addition, landmarks are differently influenced by involuntary facial movements [43].

Researchers conducting any study utilizing 3D imaging techniques should investigate their evaluation concept in advance. The revealed information (e.g., expected level of precision, reliability) needs to be discussed and taken into account in order to come to conclusions. The fact that in our study CL infants showed a lower level of accuracy compared to the non-CL infant underlines this fact.

More investigations are necessary regarding inter-observer reliability, which might be an issue for bigger studies or meta-analysis of multiple scientific studies. 

Patient-specific short- and long-term factors, such as involuntary mimicking or weight changes, must be defined regarding their level of influence [43].

The upcoming technology of 4D (video data) acquisition [35, 36] will make evaluation even more challenging. The best way to address the huge amount of information resulting from that the use of that technology will probably be a software algorithm that automatically follows landmarks through the video material.

Besides all this facts that focus on exact landmark identification and clinical study issues 3D photography in our eyes is a great tool for objective documentation of a person's facial appearance. This objective documentation is not only an important issue in the evaluation of surgical results by the means of pre-postcomparison. It also plays an important rule when medico-legal questions are raised. We believe the 3D imaging technique to be the “natural” further development of conventional photography.

## 4. Conclusions

The technical precision of 3D-soft-tissue imaging techniques, such as those provided by the 3dMDface System, are of great help in acquiring soft tissue surface data of the human face in an objective way. However, all anthropological evaluation concepts based on this data must meet high standards regarding precision and reliability. Due to the complex situation with the degree of reliability that is strictly dependent on the landmark and axis in question, we conclude that prestudies addressing the baseline accuracy of any evaluation strategy are mandatory.

Utilizing the mean of multiple measurements instead of a single measurement could clearly reduce the risk of corrupting the data during the evaluation process.

## Figures and Tables

**Table 1:** Descriptive statistics for all measured TREs; landmarks sorted by overall TRE for each landmark – best at top (all distances in mm).

	*N*	Range	Minimum	Maximum	Mean	Std. deviation
Exocanthion_R	42	,89	,04	,93	,3914	,22372
Endocanthion_R	42	1,32	,05	1,37	,4288	,27973
Subnasale	42	1,03	,05	1,08	,4486	,27415
Endocanthion_L	42	1,26	,13	1,38	,4828	,29438
Exocanthion_L	42	2,09	,04	2,13	,5054	,36775
Alar_curvature_R	42	1,42	,10	1,52	,5812	,32270
Softnasion	42	1,59	,10	1,69	,6054	,29693
Pronasale	42	1,80	,10	1,90	,6134	,40581
Crista_philtry_R	42	4,53	,07	4,59	,6338	,70541
Alare_L	42	1,89	,05	1,94	,6419	,41489
Cheilion_L	42	2,42	,12	2,54	,6815	,50383
Crista_philtry_L	42	4,22	,05	4,27	,7009	,80334
Alar_curvature_L	42	3,41	,09	3,50	,7069	,60140
Labiale_inferius	42	3,18	,03	3,21	,7148	,60663
Labiale_superius	42	4,42	,05	4,47	,8172	,94447
Stomion	30	2,46	,08	2,54	,8173	,55138
Softorbitale_R	42	1,80	,05	1,85	,8675	,42323
Alare_R	42	2,57	,18	2,74	,8691	,50198
Softporion_L	36	2,10	,12	2,22	,8975	,52477
Softpogonion	42	2,15	,12	2,27	,9124	,50013
Cheilion_R	42	6,55	,10	6,66	,9893	1,15222
Softorbitale_L	42	3,01	,05	3,07	1,0327	,61755
Softporion_R	18	3,79	,03	3,82	1,1514	,89364
Softgnathion	42	3,43	,32	3,75	1,2142	,74104
Glabella	42	2,92	,21	3,13	1,2981	,83588
Softgonion_L	29	4,87	,37	5,25	1,9057	1,34761
Softgonion_R	30	5,31	,58	5,89	2,2122	1,58298
Valid *N* (listwise)	12					
